# Extracellular vesicles in reproductive medicine: from “animal-led” to “plant-enabled”

**DOI:** 10.3389/fcell.2025.1718643

**Published:** 2025-11-21

**Authors:** Keying Pan, Wenhan Ju, Yue Wang, Qianwen Zhang, Ruyi Wang, Xingyue Jiang, Shuai Zhao

**Affiliations:** 1 First Clinical Medical College, Shandong University of Traditional Chinese Medicine, Jinan, China; 2 Guanghua Hospital, Shanghai University of Traditional Chinese Medicine, Shanghai, China

**Keywords:** extracellular vesicles, plant-derived extracellular vesicles, reproductive diseases, assisted reproduction, gametogenesis, embryonic development

## Abstract

Extracellular vesicles (EVs) have emerged as pivotal mediators of intercellular communication in reproductive medicine, demonstrating considerable potential for both understanding and treating reproductive disorders. By precisely regulating key processes such as follicular development, embryo implantation, and the immune microenvironment, EVs present innovative opportunities for the precision treatment of infertility. However, the clinical translation of conventional animal-derived EVs faces significant challenges, including inherent heterogeneity, difficulties in scalable production, and ethical concerns. These limitations have accelerated the exploration of sustainable and safer alternatives, notably plant-derived extracellular vesicles (PDEVs). This paradigm shift from animal to plant sources is paving the way for a new era of green precision medicine in EV-based therapies.

## 1 Introduction

In recent years, the global infertility rate has been on a steady rise. According to data from the World Health Organization (WHO), approximately 15%–20% of couples of reproductive age face fertility challenges. With complex etiologies and limited treatment options, this issue has become a core challenge in reproductive medicine (Inhorn and Patrizio, 2015). Although assisted reproductive technologies (such as *in vitro* fertilization, IVF) have significantly improved pregnancy outcomes for some patients, clinical live birth rates remain stagnant at 30%–40% (Glujovsky et al., 2022; Cornelisse et al., 2024), accompanied by risks like ovarian hyperstimulation, embryonic aneuploidy (Datta et al., 2021), and repeated implantation failures (Pirtea et al., 2023). To overcome this dilemma, there is an urgent need to deepen molecular-level understanding of infertility mechanisms and develop innovative diagnostic and therapeutic strategies.

Extracellular vesicles (EVs) are membrane structures actively secreted by cells, with diameters ranging from 30 to 1,000 nm. Based on their biogenesis, they can be categorized into exosomes, microvesicles, and apoptotic bodies (Nowak et al., 2023). These nanoscale carriers carry proteins, nucleic acids (such as mRNA, miRNA, lncRNA), lipids, and metabolites derived from the parent cell (Gupta et al., 2021). They participate in intercellular communication, microenvironmental remodeling, and pathophysiological regulation through paracrine or long-distance transport, becoming “molecular messengers” in the field of life sciences (Rai et al., 2024; Li H. et al., 2024). EVs, as key messengers mediating intercellular communication, have provided a novel perspective for understanding reproductive processes, every critical phase of the reproductive process—from follicular development and fertilization to embryo implantation and maternal-fetal immune tolerance—can be precisely regulated by EVs (Esfandyari et al., 2021; James et al., 2020; Jiang et al., 2023; Qu et al., 2022; Tan et al., 2022; Chen X. et al., 2023; Zhang Z. et al., 2024; Fan et al., 2021; Tsuru et al., 2024). Research has also found that changes in the content of EVs are part of promoting certain pathological conditions, making them a promising tool for diagnosis and treatment in reproductive medicine (Giacomini et al., 2020; Hadidi et al., 2023; Jahanbani et al., 2023; Ma et al., 2023; Paul et al., 2023). However, the research paradigm in this field has long been dominated by animal-derived EVs—those obtained from human body fluids or mammalian cell models (Luis-Calero et al., 2024; Mi et al., 2024; Li L. et al., 2022; Li Y. et al., 2023; Xu et al., 2025; Khalil and Altayfa, 2025; Barrachina et al., 2022; Tamessar et al., 2021). While these studies have greatly enhanced our understanding, their clinical translation faces multiple bottlenecks including limited availability, high costs, potential pathogen risks, and immune rejection challenges (Hodžić et al., 2023; Asgari et al., 2021; Zhao et al., 2021; Thabet et al., 2020). This situation has driven researchers to explore safer and more economical sources for novel EVs. In this context, plant-derived extracellular vesicles (PDEVs) have emerged as a promising therapeutic delivery platform. Derived from edible plants, PDEVs exhibit natural biocompatibility, low immunogenicity, and scalability for mass production. Preliminary studies suggest their potential as natural carriers for bioactive molecules, which could intervene in reproductive pathologies through anti-inflammatory, antioxidant, and immunomodulatory mechanisms.

This review systematically examines the strategic evolution of research paradigms in exosome studies within reproductive medicine, tracing the paradigm shift from “animal-oriented” to “plant-driven” approaches. It begins by reviewing the achievements and challenges in animal-derived exosome research, then comprehensively discusses the biological characteristics of plant-derived exosomes (PDEVs) and their potential translational value in regulating the reproductive microenvironment. Finally, we explore the prospects of PDEVs as a revolutionary tool for addressing reproductive medical challenges, while identifying the scientific and technical barriers that must be overcome before their clinical translation.

## 2 The central role of extracellular vesicles in reproductive physiology

Extracellular vesicles are now recognized as indispensable orchestrators of intercellular communication within the reproductive tract, critically regulating a continuum of events from gametogenesis to embryo implantation. Their roles extend beyond mere signal transfer to include the precise spatial and temporal regulation of key physiological processes.

### 2.1 EVs in gametogenesis

Within the ovarian follicle, a sophisticated EV-mediated cross-talk exists between the oocyte and its surrounding somatic cells. Oocyte-derived EVs (oo-EVs) deliver crucial cargo, such as miRNAs (e.g., miR-205), to granulosa cells, repressing genes like SOCS2 and thereby enhancing cell proliferation and follicular integrity (Esfandyari et al., 2021; Tan et al., 2022). Conversely, granulosa cell-derived EVs (GC-EVs) reciprocate by transporting miRNAs and proteins that regulate oocyte meiosis and metabolic homeostasis. For instance, EVs from cumulus cells carry miR-224, which targets Smad4 in oocytes to promote their maturation (Chen X. et al., 2023). This bidirectional communication ensures the synchronized development of the oocyte and its follicular microenvironment, a prerequisite for the production of a developmentally competent egg.

In the male reproductive system, EVs from Sertoli cells (Sertoli-EVs) provide vital support to developing germ cells in the seminiferous tubules, delivering nutrients, miRNAs, and proteins essential for spermatogenesis (James et al., 2020; Barrachina et al., 2022). Following testicular development, sperm undergo further functional maturation during their transit through the epididymis. This process is largely governed by epididymosomes–EVs secreted by the epididymal epithelium. Epididymosomes deliver a repertoire of macromolecules to sperm, including the fusion protein P25b/DPP9, which is crucial for sperm-egg recognition, and key enzymes like glutathione peroxidase 5 (GPX5) that protect against oxidative stress (Barrachina et al., 2022; Tamessar et al., 2021). This post-testicular modification is critical for rendering sperm functionally competent for fertilization.

### 2.2 EVs in fertilization

EVs are active participants in the final journey of sperm and the fertilization event. Follicular fluid EVs (ff-EVs) have been shown to enhance sperm motility, induce capacitation, and promote the acrosome reaction (Dorado-Silva et al., 2020; Sysoeva et al., 2021). They achieve this by transferring bioactive lipids that modify sperm membrane fluidity and by delivering regulatory molecules that modulate calcium signaling pathways. Furthermore, emerging evidence suggests that EVs from the oviductal epithelium can act as chemoattractant guides, facilitating the navigation of sperm toward the oocyte, thereby increasing the likelihood of a successful encounter.

### 2.3 EVs in early embryonic development and maternal-embryo communication

Following fertilization, EVs continue to play a pivotal role. The pre-implantation embryo itself actively secretes EVs that can influence its own development and signal its status to the maternal reproductive tract. More significantly, maternal-derived EVs from the oviduct and uterus create a supportive microenvironment for the developing embryo. Oviductal EVs contain embryotrophic factors that enhance embryonic genome activation, improve blastocyst formation rates, and regulate cell lineage specification, potentially through the modulation of key pathways like PI3K/AKT and Hippo signaling (Li Y. et al., 2023; Wang et al., 2024a). As the embryo enters the uterus, endometrial epithelial-derived EVs (EE-EVs) contribute to endometrial receptivity. A classic example is the transfer of miR-320a and miR-98 from EE-EVs to trophoblast cells, which can modulate their adhesive and invasive properties, preparing the endometrium for the incoming blastocyst (Wang X. et al., 2021). This intricate, EV-facilitated dialogue is fundamental to achieving successful implantation and establishing a pregnancy.

The precise regulation of EVs in the physiological processes outlined above underscores their fundamental role in reproductive success. However, this finely tuned system is vulnerable to disruption. Aberrations in EV biogenesis, alterations in their cargo composition (e.g., dysregulated miRNA profiles), or dysfunctional intercellular signaling can transform these vital molecular messengers into drivers of pathology. The following sections will elucidate how such dysregulation of EVs contributes to the pathogenesis of a spectrum of reproductive disorders, including polycystic ovary syndrome, endometriosis, premature ovarian failure, asthenozoospermia, and recurrent miscarriage, highlighting their dual nature as both guardians of reproductive homeostasis and agents of disease.

## 3 The role of EVs in the development of different reproductive diseases

### 3.1 The role of EVs in the development of polycystic ovary syndrome

Polycystic Ovary Syndrome (PCOS) is a common endocrine and metabolic disorder among women of reproductive age, characterized by ovulatory dysfunction, clinical and/or biochemical hyperandrogenism, and polycystic ovarian morphology. Patients often present with menstrual irregularities, infertility, hirsutism, acne, obesity, and insulin resistance. The pathogenesis of PCOS is complex, involving genetic, environmental, and metabolic factors. Studies have shown that compared with healthy people, PCOS patients have different levels of miRNA expression in follicular fluid, granulocytes and EVs in blood. These differentially expressed miRNA may be involved in the pathogenesis of PCOS, such as mediating follicle development, regulating hormone levels, insulin resistance, obesity and metabolic disorders (Jiang et al., 2023; Cao J. et al., 2022; Salehi et al., 2023).

#### 3.1.1 Follicular fluid EVs regulate ovarian granulosa cells and cumulus cells, affecting follicle development

Ovarian granulosa cells are essential for follicle development, forming the follicular wall around the oocyte and facilitating nutrient delivery to support oocyte growth and maturation, thereby ameliorating ovulation disorders (Li M. et al., 2024). Extracellular vesicles derived from oocyte follicular fluid play a critical supportive role in follicle maturation. These vesicles carry molecules such as miRNAs, mRNAs, and proteins, which participate in multiple aspects of folliculogenesis, including primordial follicle formation, oocyte development and selection, bidirectional granulosa-oocyte communication, steroid hormone regulation, and modulation of cumulus cell density, viability, proliferation, and apoptosis. Through these mechanisms, follicular fluid EVs significantly influence oocyte development, maturation, and release (de Ávila et al., 2020; Inoue et al., 2020). Intercellular communication within the follicular microenvironment is vital for oocyte growth and maturation. A thorough understanding of EV cargo and signaling pathways will further elucidate the mechanisms governing follicle development and oocyte maturation.

In polycystic ovary syndrome (PCOS) patients, follicular fluid EVs are enriched in S100-A9, which promotes inflammation and disrupts steroidogenesis by activating the NF-κB pathway, thereby impairing granulosa cell function (Li et al., 2020). Several miRNAs within EVs are dysregulated in PCOS. For instance, miR-424-5p suppresses granulosa cell proliferation and induces senescence *via* the CDCA4-mediated Rb/E2F1 pathway (Yuan et al., 2021). MiR-143-3p targets BMPR1A and inhibits Smad1/5/8 signaling, promoting granulosa cell apoptosis (Zhao et al., 2022). Conversely, miR-10b-5p enhances the secretion of chemokines CCL2 and CXCL8 by targeting BDNF in granulosa cells, thereby supporting ovulation (Yuan et al., 2023). Additionally, the lncRNA LIPE-AS1 is highly expressed in PCOS follicular fluid EVs and modulates steroid metabolism, granulosa cell proliferation, and apoptosis through the LIPE-AS1/miR-4306/LHCGR axis, contributing to poor oocyte quality and impaired embryo development (Yu et al., 2024). Thus, EV-associated miRNAs in follicular fluid likely contribute to PCOS pathogenesis by altering granulosa cell functions, though their regulatory mechanisms require further exploration.

Cumulus cells extend transzonal projections that penetrate the zona pellucida, forming the cumulus–oocyte complex. These structures enable the transfer of ions, metabolites, amino acids, and regulatory molecules *via* gap junctions, supporting bidirectional communication essential for follicular development and function (Martinez et al., 2023; Richani et al., 2021). Follicular fluid EVs not only facilitate this communication but also promote cumulus expansion, directly influencing follicle maturation. Mesenchymal stem cell (MSC)-derived EVs deliver miR-323-3p, which enhances proliferation and inhibits apoptosis of cumulus cells in PCOS by targeting PDCD4 (Zhao et al., 2019). Moreover, miR-323-3p is downregulated in cumulus cells of PCOS patients. Restoring its expression targets insulin-like growth factor 1 (IGF-1), improving steroidogenesis and reducing apoptosis in PCOS cumulus cells (Wang et al., 2019). These findings suggest that EVs in follicular fluid play a significant role in regulating follicle development and are likely involved in the pathogenesis of PCOS.

#### 3.1.2 EVs mediate cytochrome P450 enzyme synthesis and affect hormone levels

Hyperandrogenism is closely associated with cytochromeP450(CYP450), particularly evident in the pathophysiology of PCOS (de Medeiros et al., 2021). CYP450 includes subtypes such as CYP17, CYP19, CYP21, and CYP11A, which are key enzymes in the steroid hormone biosynthesis pathway (Zhai et al., 2023). Studies on ovarian membrane cells from PCOS patients have shown increased ovarian steroid production and elevated androgen levels, primarily attributed to changes in the expression of the key enzyme CYP450 in the steroid hormone biosynthesis pathway, with the CYP11A gene serving as a potential genetic biomarker playing a major role in the pathogenesis of PCOS (Chaudhary et al., 2021). EVs can influence steroid synthesis by delivering specific signaling molecules, modulating hormone secretion in endocrine axes such as the hypothalamic-pituitary-ovarian axis, regulating hormone levels, and affecting the ovarian environment. The mRNA levels of core enzymes in the steroid synthesis pathway influenced by EVs from follicular fluid in PCOS patients increased, with elevated expression of CYP11A, CYP19A, and HSD17B2. This was accompanied by changes in hormone levels in the follicular fluid. The levels of estradiol, estradiol, and isoprenolone in the follicular fluid of PCOS patients were significantly higher, while progesterone levels decreased. This suggests that the expression of mRNA in EVs is associated with changes in hormone levels in the follicular fluid. The differential expression of mRNA in the follicular fluid induces abnormal steroid production, which may be a potential mechanism for the elevated levels of estrogen and pregnenolone in the follicular fluid of PCOS patients (Yu et al., 2021). However, since the researchers did not conduct a comprehensive analysis of steroids in the follicular fluid, further research is needed to investigate the relationship between their expression levels and androgens. Currently, there are few studies on the relationship between EVs in PCOS cells and hyperandrogenism, and more research is needed in different cohorts in the future.

#### 3.1.3 EVs regulate the insulin pathway and liver metabolism, affecting insulin resistance

Insulin resistance refers to the decreased sensitivity of the body to insulin, leading to a reduced efficiency in insulin’s promotion of glucose uptake and utilization. The liver plays a crucial role in insulin metabolism and glycogen synthesis and degradation. EVs can influence the effect of insulin in the liver by regulating metabolic activities of hepatocytes. Specifically: on one hand, EVs from obese individuals may carry miRNAs and other molecules that regulate insulin signaling, affecting the reduced sensitivity of target cells to insulin; on the other hand, these EVs may also affect the metabolic activities of the liver and adipocytes, thereby modulating insulin resistance and metabolic disorders. Insulin resistance is prevalent among PCOS patients and is closely associated with their reproductive and metabolic complications (Zhao et al., 2023). The liver is the largest organ providing immune and metabolic functions, and its ectopic lipid accumulation has been widely recognized as one of the factors potentially influencing PCOS metabolic syndrome, despite ongoing debates about the causal relationship between hepatic fat deposition and hepatic insulin resistance in recent years, the correlation between the two is undeniable (Li M. et al., 2022; Rocha et al., 2017). Liver tissue and EVs from mice at different stages of PCOS, revealing complex metabolic interactions between liver tissue and EVs, where the downregulation of glycolysis and the tricarboxylic acid cycle may be related to the hepatic pathophysiology of PCOS, independent of age (Gao et al., 2022). In the PCOS rat model induced by dehydroepiandrosterone, EVs from adipose derived mesenchymal stem cells (AMSC) and their derived EVs can be partially mediated by miR-21-5P, regulating liver glucose homeostasis by targeting B cell translocation gene 2 (BTG2), significantly alleviating multiple phenotypes of PCOS rats, including metabolic abnormalities, polycystic ovaries, and infertility (Cao M. et al., 2022). These studies indicate that EVs can regulate the metabolic processes of organs and peripheral tissues, including the liver and adipose tissue, by modulating gene expression, thereby affecting insulin resistance and mediating the development of PCOS.

#### 3.1.4 EVs regulate sugar and lipid metabolism and affect obesity

Obesity promotes the development of PCOS, which in turn exacerbates obesity (Teede et al., 2023). This interaction not only affects women’s reproductive health but can also lead to a series of metabolic syndromes, such as diabetes, hypertension, and hyperlipidemia, increasing the risk of cardiovascular diseases and certain cancers, posing a serious threat to women’s overall health (Nichols et al., 2024). Excessive accumulation of fat tissue is the most prominent feature of obesity. After excluding the influence of food intake, EVs derived from brown adipose tissue in young healthy mice could reduce the body weight of obese mice induced by high-fat diet, lower blood glucose levels, and alleviate lipid accumulation, among other metabolic syndrome symptoms (Zhou et al., 2020). Overexpression of miR-20b-5p and miR-106a-5p in serum EVs in insulin-resistant PCOS mouse models could reduce adipocyte differentiation, thereby alleviating lipid metabolism disorders during the process of PCOS development caused by insulin resistance (Hong et al., 2022). In summary, EVs influence obesity by mediating glycolipid metabolism, initially revealing the significant role of EVs in regulating lipid metabolism disorders in PCOS.

### 3.2 The role of EVs in the development of endometriosis

Endometriosis (EMs) is a condition characterized by the presence of endometrial tissue (both glands and stroma) with growth function outside the uterine cavity, beyond the endometrial lining and myometrium. Its main symptoms include progressively worsening dysmenorrhea, chronic pelvic pain, dyspareunia, and infertility, which significantly impacts patients’ quality of life. The pathogenesis of EMs remains incompletely understood, with Sampson’s theory of retrograde menstruation being widely accepted. EVs play multiple roles in EMs, including promoting angiogenesis, modulating inflammation and immune responses, facilitating cell proliferation and inhibiting apoptosis, as well as participating in fibrosis formation (Freg et al., 2021). Additionally, EVs hold promise as non-invasive diagnostic markers and potential targets for targeted therapy in EMs (Esfandyari et al., 2021). As research into the mechanisms of EVs in EMs deepens, it is believed that future advancements will bring new breakthroughs to the diagnosis and treatment of EMs.

#### 3.2.1 EVs affect neurovascular angiogenesis in endometriosis

The development and progression of EMs are closely related to neurovascularization (Saunders and Horne, 2021). According to the theory of retrograde menstruation by Sampson (Sampson, 1927), endometrial tissue travels backward through the fallopian tubes and deposits in the peritoneal cavity, where it establishes a blood supply and proliferates, forming clinical EMs lesions. EVs can act as “mediators” for signal transmission, guiding vascular and neural generation through their “cargo,” promoting the survival and growth of ectopic lesions, thus playing a crucial role in the development and progression of EMs. EVs promote the proliferation, migration, and invasion of endometrial stromal cells (ESCs), inhibit ESCs apoptosis, and enhance the progression of endometriosis and angiogenesis by regulating the miR-761/histone deacetylase 1 (HDAC1) axis and activating the signal transduction and activator of transcription 3(STAT3)-mediated inflammation (Zhang et al., 2022). EVs secreted by ESCs from EMs patients can be absorbed by dorsal root ganglia, promoting nerve growth and thereby facilitating EMs angiogenesis (Sun et al., 2019). Currently, there are few reports on the relationship between EVs and neurovascularization in EMs,which requires further investigation.

#### 3.2.2 EVs regulate immune and inflammatory responses in endometriosis

Recent studies have found that the occurrence and development of EMs are closely related to immune mechanism imbalance and chronic inflammation, with EVs having a certain association with immune system imbalance and chronic inflammation. Menstrual blood flows backward through the fallopian tubes *via* uterine contractions into the peritoneal cavity. Once it enters the peritoneum, this endometrial tissue can adhere to the peritoneal structure, forming a blood supply and developing into endometriosis (Sampson, 1927). However, about 90% of women of childbearing age experience menstrual blood reflux, while the incidence of EMs is only around 10%. This suggests that women with EMs may have dysfunctional immune responses to the refluxed endometrial fragments, preventing their clearance by the immune system and ultimately promoting inflammation, angiogenesis, and pathological conditions at the site of ectopic lesions (Crump et al., 2024). The most critical factor in disease progression associated with immune imbalance is the overactivation of the immunosuppressive microenvironment. Suppressor cells derived from myeloid-derived suppressor cells (MDSCs) have been identified as one of the strongest immunosuppressive cells. EVs miRNAs derived from MDSCs may promote the occurrence and development of EMs by suppressing the body’s immune function, indicating that EVs play a significant role in the immune imbalance process of EMs (Chen Y. et al., 2019). These vesicles can transport their “cargo” to the pelvis and abdomen and deliver it to receptor cells (such as macrophages in the abdominal cavity), affecting the functional activities of receptor cells and thus exerting their effects. Thus, EVs can affect EMs immune imbalance and chronic inflammation, but due to the lack of large sample data, further research is needed.

### 3.3 The role of EVs in the development of premature ovarian failure

Premature Ovarian Failure (POF), now more commonly referred to as Primary Ovarian Insufficiency (POI), refers to the loss of ovarian function in women before the age of 40. Its clinical features include amenorrhea, decreased estrogen levels, elevated gonadotropin levels, accompanied by varying degrees of perimenopausal symptoms (such as hot flashes, night sweats, mood swings, *etc.*) and infertility. Etiological factors include genetic, immune, iatrogenic, and environmental elements. EVs play a crucial role in POF, with functions including promoting hormone secretion, facilitating angiogenesis, combating oxidative stress, and regulating apoptosis (Qu et al., 2022; Ren et al., 2023). Recent studies have shown that EVs are playing an unprecedented role in mediating cellular communication within mammalian follicles. As research on EVs deepens and technology advances, they hold promise as biomarkers for POF. It is believed that extracellular vesicle therapy will bring new hope to patients with POF (Esfandyari et al., 2021).

#### 3.3.1 EVs affect the function of ovarian granulosa cells and promote hormone secretion

Granulosa cells are the primary cell type in follicles, supporting their formation and development. They also secrete gonadotropins to maintain ovarian function, serving as a keyfor maintaining normal follicle development (Telfer et al., 2023). Atresia follicle is an important feature of POF, which is mainly caused by the apoptosis of oocyte cells, especially granulosa cells (Zhou S. et al., 2022). Multiple studies have shown that EVs can improve ovarian function through multiple pathways, including regulation of phosphoinositide 3-kinase (PI3K), SMAD. EVs derived from human umbilical cord mesenchymal stem cell (HUCMSCs) carry miR-126-3p, which targets the upregulation of the PI3K/AKT/mTOR pathway to promote angiogenesis in POF and reduce apoptosis in primary rat ovarian granulosa cells (OGCs) (Qu et al., 2022). EVs derived from follicular fluid target the inhibition of the 10th chromosome homologous deletion phosphatase and tensin homolog deleted on chro-mosome ten (PTEN), *via* miR-18b-5p, thereby activating AKT to open the PI3K signaling pathway and promote steroid synthesis (Zhou Z. et al., 2022). EVs derived from human adipose mesenchymal stem cells (HAMSCs) can inhibit the expression of Fas/FasL, caspase-3, and caspase-8 by activating the SMAD pathway (Huang et al., 2018). Extracellular vesicles derived from blood cells also play a role in oocyte development and maturation. By delivering signaling molecules such as growth factors and hormones, they influence meiosis and developmental potential of oocytes. Extracellular vesicles generated from menstrual blood stromal cells (MenSCs) deliver platelet-derived growth factor receptor-1 (PDGF-R1), which regulates the SMAD3/AKT/MDM2/P53 signaling pathway. This mechanism improves granulosa cell apoptosis and prevents premature ovarian failure (Song et al., 2023). Extracellular blood plasma vesicles and miRNA contained in them affect the hormone synthesis and proliferation of granulosa cells, among which miR-126-3p plays the most significant role. MiR-126-3p inhibits the proliferation of granulosa cells by inhibiting PDGFRβ and its downstream PI3K-AKT pathway, thus affecting the estrous cycle and hormone secretion in mice (Jiang et al., 2023).

These vesicles can also increase the mRNA and protein expression of SMAD2, SMAD3, and SMAD5 both *in vivo* and *in vitro*, thereby promoting the proliferation of granulosa cells and inhibiting their apoptosis, improving ovarian function in mice with ovarian insufficiency. Based on these studies, we found that the regulation of ovarian granulosa cells by EVs mainly manifests in two aspects: the proliferation and apoptosis of granulosa cells themselves and the steroidogenesis function of granulosa cells. The differences in regulation may be influenced by the source of the EVs and vary depending on the different miRNAs they carry.

#### 3.3.2 EVs affect ovarian angiogenesis

Ovarian function depends on the establishment and continuous remodeling of a complex vascular system. A normal vascular network is essential for supplying follicles and the corpus luteum with sufficient oxygen, nutrients, and hormones. Therefore, vascular formation is crucial for follicle development, dominance, and ovulation. Vascularogenesis is a complex biological process involving interactions between vascular-forming cells and the extracellular environment, and miRNAs are key regulators of endothelial cell function, particularly in vascularogenesis (Kumari et al., 2021). Currently, there has been extensive research on the regulatory mechanisms related to vascular formation involving EVs. In an experiment where human umbilical vein endothelial cells (HUVECs) were cultured *in vitro*, conditioned medium from mesen-chymal stem cells (MSCs) has angiogenic potential and found that EVs derived from MSCs can carry various miRNAs associated with angiogenesis and transfer these miRNAs to endothelial cells, thereby promoting angiogenesis (Wang W. et al., 2021). Additionally, EVs derived from human amniotic epithelial cells (HAECs) can inhibit acute vascular injury induced by chemotherapy in POF models, thus protecting ovarian vessels from damage (Zhang et al., 2019).

#### 3.3.3 EVs regulate ovarian antioxidant stress function

Oxidative stress is one of the primary causes of cellular aging, and exposure to toxic substances and ionizing radiation can both lead to oxidative stress (Hajam et al., 2022). Although the role of oxidative stress in the pathogenesis of POF has not been thoroughly studied, existing results indicate that ovarian oxidative stress is a cause of premature follicular failure (Cai et al., 2021). Studies have shown that nuclear factor erythroid-2-related factor 2 (Nrf2), as an oxidoreductase-sensitive transcription factor, plays a crucial role in protecting organisms from damage caused by various oxidants and toxic substances (Sies and Jones, 2020). Nrf2 and its mediators carried by EVs can regulate the antioxidant levels of target cells, thereby inducing tissue repair and regeneration (Linna-Kuosmanen et al., 2021). In an experiment using hydrogen peroxide to induce oxidative stress in granulosa cells, EVs derived from granulosa cells contain Nrf2mRNA, an mRNA that can activate antioxidant signaling pathways in other cells, particularly oocytes, thus resisting the oxidative stress environment (Saeed-Zidane et al., 2017). In addition, EVs derived from human embryonic stem cells (HESCs) can reverse the aging of endothelial cells and their ability to proliferate, migrate, and form blood vessels. This mechanism may be due to the high enrichment of miR-200a in EVs derived from HESCs, which downregulates Keap1, a negative regulator of Nrf2 expression, thereby playing a crucial role in the angiogenic process mediated by these vesicles (Chen B. et al., 2019). Inducing antioxidant responses is a promising therapeutic strategy for overcoming aging, but many unknown pathways of extracellular vesicle miRNAs in this process remain to be further investigated and confirmed.

### 3.4 The role of EVs in the development of asthenozoospermia

Asthenozoospermia (AS) is one of the common causes of male infertility, characterized by a lower proportion of progressively motile sperm in semen parameters than the reference limit. Reduced sperm motility directly impairs sperm transport through the female reproductive tract, capacitation, and the ability to bind with the oocyte, resulting in difficulties achieving conception. Sperm EVs mainly originate from the testes, epididymis, and prostate, known as testicular vesicles, epididymal vesicles, and prostatic vesicles, respectively. They play multiple roles such as regulating sperm maturation and motility, participating in sperm capacitation and acrosome reaction, and serving as biomarkers (Vickram et al., 2021). Under transmission electron microscopy, EVs secreted by Sertoli cells can be observed around all levels of germ cells, which helps regulate the growth and development of germ cells. In AS, sperm motility is significantly reduced, affecting sperm quality. We infer that the secretion of sperm EVs in AS patients may be disordered.

#### 3.4.1 EVs transport bioactive substances and regulate sperm maturation and movement

A large number of EVs exist in human semen, which can regulate the maturation and movement of sperm. However, in AS patients, the secretion and function of EVs in semen may be disrupted, leading to reduced sperm motility. The membrane proteins of prostatic bodies increase intracellular Ca2+ levels in sperm cells by regulating calcium channels, enhancing sperm motility. Transfer proteins (such as galactocystin-3 and CD48) modulate immune response pathways in the female reproductive tract to protect sperm from being cleared by the female immune system and induce superactivation of sperm, playing a crucial role in sperm movement and fertilization (Vickram et al., 2020). Adding prostatic bodies to culture media can enhance the vitality of sperm in AS patients and cryopreserved sperm, with a positive correlation to concentration (Saadeldin et al., 2020).

When sperm pass through the female reproductive tract, they interact with extracellular vesicles from different sites. Follicular fluid extracellular vesicles play a crucial role in regulating sperm function. These vesicles enhance the fertilization capability of sperm, influence their movement trajectory and speed, enabling them to better adapt to the needs of the fertilization process. They improve the sperm’s ability to penetrate the cumulus cells and increase resistance to zona pellucida hydrolysis, making the zona harder and reducing multiple sperm fertilization. Additionally, they boost sperm vitality, regulate receptor expression on the sperm surface, and promote recognition and union between sperm and ovum (Dorado-Silva et al., 2020). By analyzing the effects of extracellular vesicles from follicular fluid on sperm motility, it will help improve the sperm quality of patients with asthenozoospermia and increase the fertilization ability of sperm, so as to improve the treatment effectiveness of male infertility factors (Sysoeva et al., 2021).

#### 3.4.2 EVs participate in intercellular communication and affect sperm capacitation and acrosome reaction

Extracellular vesicles (EVs) provide novel insights into intercellular communication by demonstrating the transfer of key bioactive molecules—including proteins, lipids, DNA, mRNA, microRNA, circular RNA, and long non-coding RNA—between the epididymis and sperm (Ali et al., 2023). Seminal vesicle EVs contain lipids such as ceramide, cholesterol, phosphatidylserine, glycerol phosphate, and sphingomyelin, which contribute to vesicle stability, facilitate intercellular signaling, and influence sperm capacitation and the acrosome reaction (Brouwers et al., 2013). These EVs encapsulate lipids and other bioactive components within their membrane structure, delivering them to target cells through direct intercellular contact or *via* bodily fluids.

During capacitation, cholesterol efflux from the sperm membrane increases membrane fluidity and induces the redistribution of surface proteins, activating essential signaling pathways (Nicolli and Cesari, 2023). Concurrently, membrane fluidity is modulated to prevent premature capacitation (Tamessar et al., 2021). EVs also convey signaling molecules such as glutathione peroxidase-5 (GPX5) and prostaglandins, which promote sperm capacitation and modulate immune responses within the female reproductive tract, thereby establishing a favorable microenvironment for fertilization (Huang J. et al., 2024). Additionally, EV-associated proteins and enzymes regulate the acrosome reaction by facilitating the release of acrosomal enzymes and disrupting the acrosomal membrane during sperm–oocyte interaction, thus enhancing sperm penetrative ability (Roy et al., 2023). Epididymosomes further deliver numerous proteins to sperm, including enzymes, chaperones, and structural proteins like sperm adhesion molecule 1, which participates in zona pellucida binding, prevents premature acrosome reaction, and protects against oxidative damage (Barrachina et al., 2022).

When sperm traverse the female reproductive tract, endometrial cell-derived EVs interact with the sperm surface. They modulate sperm membrane fluidity, remove surface coatings, and alter membrane permeability, thereby improving the ability of sperm to penetrate the cumulus cell layer. These EVs also help attenuate local immune responses, reducing immune-mediated sperm damage and supporting sperm survival. Collectively, these modifications promote sperm capacitation and facilitate zona pellucida penetration. Studies have shown that brief co-incubation of sperm with endometrial EVs enhances the acrosome reaction, elevates reactive oxygen species (ROS) levels, and stimulates protein tyrosine phosphorylation, all indicative of capacitation. These effects are particularly pronounced under stimulation by calcium ionophores (Franchi et al., 2016; Deng et al., 2024; Murdica et al., 2020).

#### 3.4.3 Extracellular vesicles carry genetic material and affect embryo quality and development

Sperm extracellular vesicles contain a large amount of mRNA and potential small non-coding RNAs (sncRNA), such as miRNAs. These RNAs can reflect the pathophysiological status of their source cells. Researchers once thought they were merely residual products of spermatogenesis with no biological function, but growing evidence suggests that specific sperm RNAs play a crucial role in fertilization and epigenetics of embryos (Candenas and Chianese, 2020). For example, extracellular vesicles in human semen interact with T cells *in vitro* and drive their differentiation into regulatory T cells, inducing immune tolerance to paternal allogeneic antigens in the female genital tract and during fetal development (Zhang X. et al., 2024).

The Developmentai Origins of Health and Disease (DOHaD) suggests that the environment in which a fetus is exposed can influence susceptibility to disease and the risk of developing it in adulthood. Recent studies have increasingly shown that sncRNA carried by mammalian sperm can transmit paternal environmental influences (such as metabolic diseases, psychological trauma, and exposure to toxic substances) to offspring, affecting their health (Heard and Martienssen, 2014; Takahashi et al., 2023). Sperm lacks complete organelles and cannot achieve *de novo* transcription. Therefore, the RNA transport function of extracellular vesicles in sperm plays an important role in establishing sperm epigenome, which then affects embryonic development and ultimately leads to the transgenerational transmission of epigenetic effects by environmental effects (Ashapkin et al., 2023). Therefore, we speculate that environmental exposure leads to changes in seminal extracellular vesicle RNA that can transmit adverse information to offspring. This may help us analyze the mechanisms of disease that cannot be fully explained by genetic factors, such as obesity and type 2 diabetes.

#### 3.4.4 EVs have potential value in the diagnosis and treatment of asthenozoospermia

EVs can serve as biomarkers for AS diagnosis. By analyzing the components of seminal EVs, such as proteins, lipids, and nucleic acids (especially miRNAs), which reflect the physiological state and pathological changes of sperm, it is possible to detect impaired spermatogenesis in males, thus providing crucial evidence for the diagnosis of AS. Protein in different diseases has differential expression. Through extracellular vesicle purification technology and proteomics tools, 1,474 extracellular vesicle proteins were identified in normal semen extracellular vesicles, which are mainly related to protein metabolism, cell growth and maintenance. Abnormal appearance of these proteins will seriously affect reproductive ability, which is an effective basis for becoming a biomarker (Candenas and Chianese, 2020; Yang et al., 2017). In patients with AS, the expression of miR-345-3p is upregulated while that of miR-2412 is downregulated in seminal EVs, suggesting that these may influence downstream related proteins through regulation of target genes, thereby affecting sperm motility (Taravat et al., 2024). Additionally, comparing the expression of miRNA in seminal EVs between patients with normal and impaired spermatogenic function revealed high sensitivity and specificity for diagnosing oligozoospermia, and also helped predict whether individuals with severe spermatogenic disorders had residual spermatogenesis (Larriba et al., 2024). The DNA in extracellular vesicles of seminal cells originates from various pathways, such as small fragments of DNA generated during the repair of genomic DNA damage or fragment DNA produced when cells are near apoptosis. These DNA fragments are encapsulated within the membrane of the vesicle or adsorbed on its outer surface. They achieve specific recognition and binding to target cell receptors through interactions with specific proteins or lipids on the membrane, thereby facilitating the transport of DNA and participating in intercellular signaling and functional regulation (Kowalczyk et al., 2022). DNA in extracellular vesicles of seminal cells has potential value as a biomarker for the diagnosis of reproductive system diseases such as male infertility (Khodamoradi et al., 2022). By studying the types and quantities of extracellular vesicle transport components in semen, we can gain a deeper understanding of the function and regulation mechanism of male reproductive system, and provide new ideas and methods for the diagnosis and treatment of infertility.

However, although EVs show great potential in the diagnosis and treatment of asthenozoospermia, they are still in the research stage. Before their application in clinical practice, further studies are needed to verify their safety and effectiveness, and reasonable ethical and legal norms should be formulated to guide the clinical use of EVs.

### 3.5 The role of EVs in the development of recurrent miscarriage

Recurrent Miscarriage (RM) refers to the occurrence of two or more consecutive spontaneous abortions with the same partner. The etiology is complex, involving factors such as embryonic chromosomal abnormalities, maternal endocrine disorders, uterine anatomical abnormalities, immune factors, and thrombophilic disorders. RM causes significant psychological distress to patients and remains a considerable challenge in the field of reproductive medicine (van Wely, 2023). Exosomes play a significant role in recurrent miscarriage RM by modulating immune responses, affecting endometrial function, and participating in abnormal embryonic development. As research advances and technology continues to evolve, exosomes hold promise as new targets and methods for diagnosing and treating recurrent miscarriage.

#### 3.5.1 EVs are involved in immune regulation and inflammatory response

In patients with RM, immune system abnormalities and excessive inflammatory responses are among the key causes. Exosomal vesicles can influence the function of immune cells such as dendritic cells and T lymphocytes by carrying immunomodulatory molecules and anti-inflammatory factors, thereby suppressing overreaction of the immune system. They can also regulate the maternal-fetal immune tolerance state through specific pathways, thus maintaining pregnancy stability. In animal experiments that exosomal vesicles can modulate the function of T cells and macrophage activity at the maternal-fetal interface, leading to reduced embryo absorption rates and decreasing the likelihood of RM (Xiang et al., 2020). Platelet-derived purified extracellular vesicle product (PEP) can induce the proliferation and wound healing of human endometrial cells, and has a regenerative effect on human endometrial cells (Miller et al., 2022). Circulating extracellular vesicles from fertile animals did not alter endometrial mediator gene expression, whereas circulating extracellular vesicles from infertile animals enhanced endometrial expression of inflammatory mediators, which may lead to abnormal inflammation, affect endometrial remodeling, and reduce fertility (Koh et al., 2020). Through animal experiments that extracellular vesicle miR-331 reduces NF-κB p65 phosphorylation by inhibiting the Notch1/IKKα pathway, thereby suppressing macrophage activation and slowing disease progression in endometritis mice (Jiang et al., 2024). At the same time, exosomal vesicles can carry inflammatory factors, enhancing inflammatory responses through intercellular communication, altering the endometrial environment, and increasing the risk of recurrent miscarriage. For example, plasma exosomal vesicle levels are elevated in patients with antiphospholipid syndrome, activating endothelial cells, exhibiting pro-inflammatory and pro-coagulant effects, directly interacting with cell receptors, and transferring biomaterials to form thrombotic emboli, promoting the development and progression of RM (Štok et al., 2021).

#### 3.5.2 Extracellular vesicles participate in the regulation of endometrial cell epithelial mesenchymal transformation (EMT)

Under normal circumstances, the epithelial cells of the endometrium maintain their specific structure and function, providing a suitable environment for embryo implantation. However, if abnormal EMT occurs, it can lead to the loss of polarity and adhesion of the endometrial epithelial cells, making intercellular connections loose. This change affects the endometrium’s receptivity to embryos, reducing the success rate of embryo implantation. Moreover, abnormal EMT may also trigger inflammatory responses and fibrosis in the endometrium, further interfering with the normal implantation and development of embryos. Studies have shown that extracellular vesicles derived from the endometrium and the miRNAs they carry can influence cellular EMT, which is closely related to the occurrence and progression of RM, affecting pregnancy outcomes (Li et al., 2018). Through animal experiments that extracellular vesicle miR-22-5p_R-1 inhibits the metabolic shift from mitochondrial respiration to glycolysis (MGS) in the trophoblast and suppresses trophoblastic EMT, increasing the likelihood of RM (Xiong et al., 2024). Compared to the control group, miR-146b-5p was significantly upregulated in patients with RM, and miR-146b-5p can reduce the invasiveness of trophoblasts by acting on MMP-6, leading to the development of RM. In summary, extracellular vesicles play a complex and important role in the development of recurrent miscarriage (Qin et al., 2016). As research on extracellular vesicles continues to advance, we hope to provide more effective diagnostic and therapeutic strategies for patients with RM.

#### 3.5.3 Participate in embryo implantation

**FIGURE 1:**
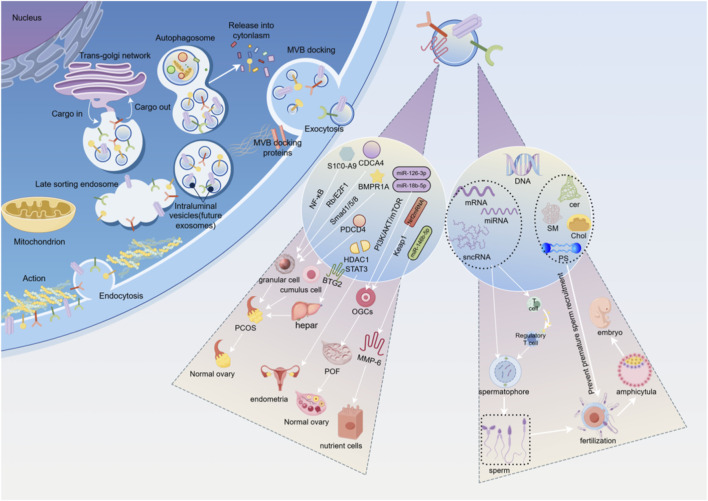
This is an original image created with Figdraw. (1) Left side of the intracellular process diagram. (2) Intermediate regulatory pathway and cell type diagram. The EVs in the follicular fluid of PCOS patients are rich in S100-A9, which significantly exacerbates inflammation and disrupts steroidogenesis by activating the NF-κB signaling pathway, playing a vital role in regulating ovarian granulosa cell function. MiRNA is differentially expressed in the EVs of follicular fluid from PCOS patients and inhibits ovarian granulosa cell proliferation and induces ovarian granulosa cell senescence by blocking the Rb/E2F1 signaling pathway mediated by CDCA4. MiRNA from follicular fluid EVs in PCOS targets the recombinat BMPR1A and blocks the Smad1/5/8 signaling pathway, promoting apoptosis of ovarian granulosa cells. EVs derived from MSCs, promote cell proliferation and inhibit apoptosis in cumulus cells by targeting the programmed cell death effector PDCD4 in the PCOS. In the PCOS rat model induced by dehydroepiandrosterone, EVs from AMSC and their derived EVs can be partially mediated by miR-21-5P, regulating liver glucose homeostasis by targeting BTG2, significantly alleviating multiple phenotypes of PCOS rats, including metabolic abnormalities, polycystic ovaries, and infertility. EVs promote the proliferation, migration, and invasion of ESCs, inhibit ESCs apoptosis, and enhance the progression of endometriosis and angiogenesis by regulating the HDAC1 axis and activating the STAT3-mediated inflammation. EVs derived from HUCMSCs carry miR-126-3p, which targets the upregulation of the PI3K/AKT/mTOR pathway to promote angiogenesis in POF and reduce apoptosis in primary rat OGCs. EVs extracted from follicular fluid activated AKT by miR-18b-5p, opened PI3K signaling pathway, and promoted steroid synthesis. Nrf2mRNA plays a key role in mediated ovarian angiogenesis by downregulating Keap1. MiR-146b-5p can reduce the invasiveness of trophoblasts by acting on MMP-6, leading to the development of RM. (3) Right side exosome contents and reproductive process diagram. Sperm extracellular vesicles (EVs) primarily originate from the testis, epididymis, and prostate, and are accordingly classified as testicular vesicles, epididymal vesicles, and prostatic vesicles. As multifunctional nanocarriers, they transport proteins, nucleic acids (such as DNA, mRNA, miRNA, lncRNA), lipids (cer, SM, Chol, PS), and metabolites derived from parental cells. They play essential roles in regulating sperm maturation, motility, capacitation, and the acrosome reaction, thereby facilitating sperm-egg binding, improving fertilization rates, and supporting the formation of healthy embryos.

Molecules like miRNAs in EVs can regulate gene expression in the endometrium, promoting the proliferation of trophoblast cells, affecting the receptivity of the endometrium, and regulating the apoptosis and adhesion of blastocysts, thereby facilitating successful implantation of the embryo in the uterus. Additionally, EVs serve as a crucial medium for intercellular communication, capable of transmitting signaling molecules from the follicular microenvironment, coordinating cell interactions during follicle development and embryo implantation. Ovum fluid is composed of secretions from the ovarian cortex and plasma exudates, serving as an important body fluid in reproduction. The EVs derived from ova fluid can carry factors that promote embryonic development, such as growth factors. These factors can act on the embryo during its migration from the fallopian tube to the uterus, promoting its development and implantation (Wang et al., 2023). Endometrial EVs carry bioactive substances such as vascular growth factors, inflammatory factors, and miRNAs, which can regulate blood flow in the endometrium, modulate inflammatory conditions, influence the regeneration and repair of damaged endometrium, and mediate the transfer of bioactive substances between endometrial cells and embryonic cells through intercellular communication, thereby affecting the implantation and development outcomes of embryos. A decrease in miR-218 levels in EVs derived from endometrial epithelial cells can impair embryo development and reduce placental trophoblast cell migration by targeting secreted frizzled related protein 2 (Wang X. et al., 2021). EVs can increase the ratio of inner cell mass to trophoblast cells during in vitro embryo culture, reduce the rate of blastocyst apoptosis, and thus enhance the embryo's implantation rate (Wang et al., 2024a). See Figure 1.

## 4 Research progress of extracellular vesicles from plant sources in reproductive medicine

In recent years, EVs have been awarded the title of “natural nanomedicine” due to their natural targeting, low immunogenicity and biobarrier penetration ability, which has set off a research boom in regenerative medicine and disease treatment (Li H. et al., 2024). In reproductive medicine, EVs have emerged as a game-changing therapeutic approach for infertility by precisely regulating critical processes including follicular development, embryo implantation, and immune microenvironment modulation. However, the inherent challenges of traditional animal-derived EVs—such as heterogeneity, scalability issues, and ethical concerns—have driven researchers to explore more sustainable and safer alternatives—Plant-derived extracellular vesicles (PDEVs) (Lian et al., 2022; Kürtösi et al., 2024). This paradigm shift from “animal-derived to plant-based” heralds EVs therapy entering a new era of green precision medicine, with extracellular vesicle (EVs) treatment transitioning from “animal-centric” approaches to a “plant-powered” phase. By integrating single-cell sequencing, synthetic biology, and AI screening technologies, we aim to establish an integrated platform for PDEVs that combines “active component mapping-target prediction-precision delivery” (Zeng et al., 2024), and provide green and efficient therapeutic solutions for reproductive disorders such as premature ovarian failure, recurrent implantation failures, and spermatogenic dysfunction (Shao et al., 2023; Cao et al., 2025), It provides theoretical blueprint and strategic inspiration for nanoscale precision intervention in reproductive medicine.

PDEVs are nanoscale lipid bilayer vesicles secreted by plant cells, carrying bioactive molecules such as proteins, nucleic acids, lipids and metabolites. Recent studies have found that PDEVs show great potential in regulating mammalian cell functions across species, opening up a new research direction in the field of reproductive medicine (Urzì et al., 2022; Zhu et al., 2020).

### 4.1 Biological properties of PDEVs

PDEVs are widely found in fruits, vegetables and herbs. Most of them are spherical or cup-shaped nanoparticles with a diameter of 30–1,000 nm, and the typical bilayer membrane structure can be seen under electron microscopy (Jackson et al., 2023; Wu et al., 2023). As a rich source of plant-specific active substances, its core components include nucleic acids (miRNA, siRNA and mRNA fragments), proteins (metabolic enzymes, heat shock proteins, membrane transporters and plant-specific proteins), lipids (phospholipids, sphingolipids, phytosterols), and secondary metabolites (flavonoids, terpenes, alkaloids and other small active molecules) (Wu et al., 2023; Xu et al., 2023), It has potential gene regulatory function, and can rely on the natural phospholipid bilayer membrane to provide good biocompatibility, low immunogenicity and anti-digestive enzyme degradation ability, protect the contents and promote transmembrane delivery.

### 4.2 Mechanism of action of PDEVs

#### 4.2.1 Intercellular communication and signal regulation

PDEVs have emerged as a promising candidate for next-generation drug delivery systems, as they can encapsulate and deliver large quantities of bioactive molecules (Mu et al., 2014; Zheng et al., 2024; Trentini et al., 2024). These bilayer vesicles, measuring 30–1,000 nm in diameter, are released by different cell types and play a crucial role in cross-cell communication between plants and humans (Calzoni et al., 2024). PDEVs carry miRNAs that can be taken up by germ cells such as oocytes, sperm, and embryonic cells, or somatic cells including granulosa cells and endometrial cells. These miRNAs regulate the expression of target genes involved in proliferation, apoptosis, and differentiation. Their surface proteins interact with host cell membrane receptors to activate or inhibit intracellular signaling pathways like MAPK, PI3K/AKT, and Wnt/β-catenin. Notably, plant-derived EVs in transgenic tobacco can communicate across species boundaries by carrying nucleic acids that downregulate IL-1β and IL-6, demonstrating their regulatory capabilities in both signaling and immune responses (Urbanelli et al., 2025). As a carrier of photoinduced metabolites that exhibit cytotoxicity, the EVs derived from aloe can lead to significant oxidative stress in melanoma cells, which can confirm the existence of intercellular communication and signal regulation (Calzoni et al., 2024).

#### 4.2.2 Antioxidant and anti-inflammatory effects

PDEVs are rich in antioxidant enzymes (SOD, peroxidase) and small molecules (flavonoids, vitamins) to effectively remove excess reactive oxygen species (ROS) in the reproductive system (Tiwari et al., 2025; Di Raimo et al., 2024; Kim M. et al., 2023; López de Las Hazas et al., 2023), To reduce oxidative stress damage to gametes (eggs and sperm) and embryos. The PDEVs mixture of grape, red orange, papaya, pomegranate and tangerine activates the Nrf2 pathway by delivering miRNAs, enhancing the antioxidant capacity of endometrial cells (Di Raimo et al., 2024). The EVs derived from mulberry can induce ROS production through the oxidizing agent DMNQ, while significantly reducing glucose oxidase-mediated ROS levels in human microvascular endothelial cells (HMEC-1). This finding suggests that PDEVs may serve dual roles: protecting the biological functions of plant-derived active compounds and acting as delivery vehicles for genetic material and functional proteins. By bridging physiological concentrations with therapeutic effects, this mechanism provides a novel molecular basis for realizing mulberry’s health-promoting potential (Garrett et al., 2024).

By inhibiting the inflammatory pathway such as NF-κB, PDEVs can downregulate the expression of inflammatory factors such as TNF-α, IL-1β and IL-6, and improve the imbalance of reproductive microenvironment caused by inflammatory diseases (such as pelvic inflammatory disease and endometriosis) or oxidative stress (Hao et al., 2024). EVs derived from papaya exhibited significant anti-inflammatory effects under LPS induction. Experimental results showed that these vesicles could reduce the production of nitric oxide (NO), downregulate the mRNA expression levels of pro-inflammatory cytokines IL-1β and IL-6, while upregulating the mRNA expression of anti-inflammatory cytokine IL-10, effectively inhibiting the migratory activity of macrophages and neutrophils (Iriawati et al., 2024). The pretreatment of lemongrass-derived extracellular vesicles (LEVs) reduced the expression of pro-inflammatory cytokines including IL-6, IL-1β, and TNF-α, while decreasing nuclear translocation and phosphorylation of NF-κB in LPS-stimulated mouse macrophages. Additionally, *ex vivo* experiments with human primary T lymphocytes demonstrated LEVs’ ability to reduce pro-inflammatory cytokines and enhance anti-inflammatory molecules, thereby confirming that LEVs overinhibit the ERK1-2/NF-κB signaling pathway to exert anti-inflammatory effects both *in vitro* and *in vitro* conditions (Raimondo et al., 2022). Lotus-derived EVs (LDEVs) were able to reduce the ability of LPS to induce inflammation in cells, thereby significantly reducing nitrite concentrations in the culture medium. In addition, LDEVs showed potential for wound healing and promoted cell migration *in vitro* (Lo et al., 2025). These results support the potential of PDEVs for use in reproductive medicine and are expected to be a component of assisted reproductive technology *in vitro* embryo culture (Yi et al., 2023).

#### 4.2.3 Improve bioavailability and targeted drug delivery

PDEVs are biological nanovesicles with a variety of bioactive functions. Due to their unique biocompatibility, low immunogenicity and good cross-species transmission ability, they have become an emerging carrier to improve bioavailability (Feng et al., 2025; Kathait et al., 2024). In the field of oral administration, they have significant advantages such as preventing gastrointestinal degradation, penetrating intestinal epithelial barrier, specific localization, safety and high yield (Fang and Liu, 2022; Alzahrani et al., 2023). The natural carrier properties of PDEVs are used to load therapeutic nucleic acids (siRNA, mRNA) or small molecule drugs for targeted delivery to reproductive organs or cells for gene therapy or precision drug delivery (Zhang J. et al., 2025; Chen Q. et al., 2023).

Evolutionary Virus-derived (KEVs) from kiwifruit possess unique liver-targeting properties. As drug carriers, they effectively reduce gastrointestinal drug leakage and enhance intestinal epithelial permeability, ultimately achieving liver-specific accumulation. The KEV-mediated drug delivery system significantly inhibits abnormal cell proliferation. This discovery provides crucial evidence for developing targeted therapeutic strategies based on PDEVs (Fang et al., 2023). Bitter melon derived extracellular vesicles (BMEVs) exert their biological effects by significantly downregulating NLRP3 inflammasome expression. Given that NLRP3 activation has been demonstrated to be closely associated with the development of cellular drug resistance, this unique mechanism suggests that BMEVs may possess potential advantages in overcoming drug resistance in oral administration systems. This discovery provides a crucial theoretical foundation for developing novel plant-derived exosome-based drug delivery systems targeting antimicrobial resistance (Yang et al., 2021). The production of grape-derived EVs (GEVs) was higher than that of tomato-derived EVs (TEVs). GEVs showed higher efficiency in loading heat shock protein 70 (HSP70) than TEVs, and higher efficiency in delivering HSP70 to cells than TEVs, showing the potential of delivering functional molecules to human cells (Kilasoniya et al., 2023). However, in the process of clinical translation, PDEVs still face many challenges, including the standardization of their extraction and characterization methods, the optimization of efficient drug loading strategies, and the study of the mechanism of action in improving the bioavailability of orally insoluble drugs, macromolecules and nucleic acid drugs (Nemati et al., 2022), The in-depth exploration of these problems will provide new research ideas and theoretical basis for the development of efficient and safe reproductive medicine drug delivery system.

#### 4.2.4 Immune regulation and microbiome regulation

Plant-derived extracellular vesicles (PDEVs) demonstrate significant immunomodulatory potential by regulating macrophage polarization toward the M2 phenotype, modulating T-cell subset proportions, and inducing immune tolerance. These mechanisms are crucial for maintaining maternal-fetal interface immune balance and preventing embryonic rejection (Li et al., 2025; Karabay et al., 2025). A notable example is Petasites japonicus-derived EVs (PJ-EVs), which significantly promote dendritic cell (DC) maturation by upregulating surface markers (CD80, CD86, MHC-I, MHC-II), enhancing Th1-polarizing cytokine secretion (TNF-α, IL-12p70), and improving antigen presentation capacity while reducing antigen uptake. PJ-EV-treated DCs effectively activate naïve T cells, promoting their differentiation into Th1 and cytotoxic CD8^+^ T cells, while stimulating IFN-γ and IL-2 secretion. These properties position PJ-EVs as a promising immunomodulatory candidate for immunotherapy development (Han et al., 2021).

PDEVs also contribute to regulating intestinal homeostasis, inflammatory responses, and metabolic disorders associated with obesity. Emerging evidence suggests that PDEVs can modulate the reproductive tract microbiota composition, indirectly influencing local immunity and the nutritional microenvironment. Although complex metabolic diseases remain challenging to cure, PDEVs offer a viable strategy for managing conditions such as obesity-related reproductive disorders (Niu et al., 2023; Emmanuela et al., 2024; Liu et al., 2025). For instance, Reconstructed Nanovesicles (Rec-tNVs) from turmeric exhibit dose-dependent lipid-lowering effects *via* multiple mechanisms: promoting lipolysis, inhibiting adipogenesis, inducing adipocyte browning, and triggering apoptosis post-endocytosis. These vesicles provide a novel weight-management strategy for PCOS patients, improving conception rates and establishing a new paradigm for plant-derived nanotherapies (Wang et al., 2024b). Similarly, mulberry bark-derived EVs (MBELN) activate the aryl hydrocarbon receptor (AhR) signaling pathway through HSPA8, alleviating colitis in mouse models. This effect involves the COPS8-mediated interorgan communication pathway, regulating inflammatory responses in microbiota-rich intestinal environments. MBELN thus represents a promising candidate for treating gut-inflammatory diseases and associated reproductive disorders such as PCOS (Sriwastva et al., 2022).

Compared to mammalian-derived EVs, PDEVs exhibit lower immunogenicity and toxicity, making them safer for therapeutic applications (Wu et al., 2023; Raimondo et al., 2022; Zuo et al., 2025). Their plant origin minimizes risks of immune reactions, while their biocompatibility supports repeated administration without significant adverse effects.

### 4.3 The prospect of conversion of PDEVs

Plant-derived exosome vesicles (PDEVs) demonstrate significant translational potential in biomedical applications due to their unique biological properties and manufacturing advantages (Mu et al., 2023). Compared with mammalian-derived extracellular vesicles, PDEVs exhibit distinct core benefits (Langellotto et al., 2025; Logozzi et al., 2022). Originating from plants, PDEVs differ in surface protein composition and lipid profiles from mammalian vesicles, significantly reducing the risk of adverse immune responses and making them more suitable for long-term or repeated administration (Karamanidou and Tsouknidas, 2021; Barzin et al., 2023). Additionally, plant biomass is abundant and cultivation conditions are relatively controllable, enabling production costs that are substantially lower than those of mammalian cell-cultured vesicles, making large-scale industrial production feasible (Li Y. et al., 2024). Furthermore, PDEVs possess a natural lipid bilayer structure that protects their payload—such as nucleic acids, proteins, and metabolites—from degradation while enhancing targeted delivery (Jin et al., 2024). The plant-based cultivation process eliminates the need for complex bioreactors or animal-derived culture media, thereby reducing ethical concerns while aligning with environmental sustainability principles (Sergazy et al., 2025). These advantages position PDEVs as a crucial translational asset in reproductive medicine, particularly for treating reproductive disorders and optimizing ART.

#### 4.3.1 New strategies for the treatment of reproductive disorders

Reproductive disorders such as premature ovarian failure, ovulation dysfunction, endometrial receptivity impairment, and reduced sperm motility have increasingly become major challenges affecting reproductive health, with current treatment options showing limitations. In recent years, PDEVs have emerged as a novel natural nanotechnology platform demonstrating significant potential. These bioactive nanocarriers deliver plant-specific miRNAs, lipids, and proteins to regulate mammalian cells across biological systems. By repairing germ cell damage and improving uterine microenvironmental conditions, PDEVs provide innovative therapeutic approaches for addressing reproductive disorders including ovarian insufficiency and embryo implantation failure. For POF—a condition characterized by reduced follicular reserve and hormonal imbalances—research has revealed that PDEVs derived from ginseng and ginger contain abundant antioxidant and anti-apoptotic compounds such as saponins and curcumin. These bioactive substances effectively reduce granulosa cell apoptosis while enhancing follicular survival (Chu et al., 2024; Kumar et al., 2022; Zhang X. et al., 2025). Specifically, PDEVs from ginseng may inhibit oxidative stress by activating the PI3K/AKT signaling pathway, thereby delaying ovarian dysfunction (Kim J. et al., 2023), Grapefruit PDEVs, on the other hand, significantly improved the ovarian microenvironment by regulating the expression of pro-inflammatory factors such as TNF-α and IL-6 with their rich flavonoids (Wang et al., 2014). In terms of endometrial repair, thin endometrium and chronic endometritis are important factors leading to embryo implantation failure. PDEVs from ginger promote angiogenesis and endometrial repair by upregulating the expression of VEGF and IGF-1 (Zhu and He, 2023), Meanwhile, grapefruit and tea-derived PDEVs inhibited NF-κB pathway through their natural anti-inflammatory components, effectively reducing endometrial inflammatory response (Iriawati et al., 2024; Chen Q. et al., 2023). For male infertility, especially in cases associated with low sperm motility, DNA damage or oxidative stress, PDEVs from pomegranate and blueberry are rich in polyphenols that scavenge ROS (Kim J. S. et al., 2023; Nguyen et al., 2023), significantly reducing sperm DNA rupture, While ginseng PDEVs enhance sperm motility by enhancing mitochondrial function (Ni et al., 2022). Together, these findings indicate that PDEVs have broad application prospects in the field of reproductive disorders treatment.

#### 4.3.2 ART optimize

In IVF and embryo culture processes, embryo quality and endometrial receptivity are critical factors for successful pregnancy. PDEVs can serve as natural and safe additives to optimize existing ART technologies. Traditional embryo culture media relying on serum or synthetic components may introduce unknown risks, whereas PDEVs can provide natural growth factors. For instance, ginseng-derived PDEVs may simulate the *in vivo* microenvironment to support early embryonic development (Shumakova and Kiselev, 2014), while antioxidant-rich PDEVs (such as those sourced from mulberries and grapefruit) can reduce ROS accumulation during culture, thereby improving blastocyst formation rates (Garrett et al., 2024; Feng et al., 2023; Savcı et al., 2021). In the process of sperm selection (e.g., density gradient centrifugation), PDEVs can protect the integrity of sperm membranes, and OFI-EVs from cactus fruits contain plant sphingolipid components that may reduce cryo-recovery damage by stabilizing sperm membrane structure (Valentino et al., 2024), Sesame-derived extracellular vesicles (SAEVs) may also improve fertilization rates by regulating calcium ion channels to promote sperm capacitation (Zhao et al., 2025). In frozen embryo transfer cycles, PDEVs can improve endometrial receptivity and promote endometrial decidualization by inducing apoptosis (Wang et al., 2025), It may also replace or supplement traditional estrogen regimens to reduce hormone dependence and reduce the risk of ovarian hyperstimulation syndrome (OHSS).

Although PDEVs have great potential in reproductive medicine, the standardized preparation of PDEVs needs to be solved. Different plants and extraction methods may affect the composition and efficacy of PDEVs, so unified quality control standards need to be established (Huang L. et al., 2024; Cui et al., 2024; Ali et al., 2022). The mechanism of PDEVs has not been fully elucidated, and more animal and clinical trials are needed to verify their safety and efficacy, as well as further exploration of their interactions with germ cells (Liu et al., 2024; Lo et al., 2024). In the future, with further research, PDEVs are expected to become a revolutionary tool for the treatment of reproductive disorders and optimization of ART, providing a safer and more efficient treatment option for infertile patients. See Table 1 for details.

**TABLE 1:** The therapeutic mechanism and potential value of PDEVs in reproductive medicine.

Exosome origin	Models/study subjects	Treatment mechanism	Treatment effect	References
Aloe	Melanoma cell	As a carrier of light-induced cytotoxic metabolites, induction significantly induces oxidative stress	To confirm its ability to mediate intercellular communication and signal regulation	(Calzoni et al., 2024)
Transgenic Tobacco	Cross-species models	Carrying nucleic acids can downregulate IL-1β and IL-6	Demonstrate cross-species communication capabilities, regulate signaling and immune responses	Urbanelli et al. (2025)
Mulberry	Human microvascular endothelial cells (HMEC-1)	1. Induced oxidative agent DMNQ-mediated ROS production 2. Reduced glucose oxidase-mediated ROS levels	Play a dual role: protect the function of plant active ingredients, and act as a delivery carrier for genetic material and functional proteins	Garrett et al. (2024)
Grape, Red Orange, Papaya, Pomegranate, Tangerine	Endometrial cell	Activate the Nrf2 pathway by delivering miRNAs	Enhance the antioxidant capacity of endometrial cells	Di Raimo et al. (2024)
Papaya	LPS induced inflammatory model	Reduce NO production, downregulate the mRNA of pro-inflammatory factors IL-1β and IL-6, and upregulate the mRNA of anti-inflammatory factors IL-10	Significantly anti-inflammatory, effectively inhibiting the migration activity of macrophages and neutrophils	Iriawati et al. (2024)
Lemon	LPS stimulated mouse macrophages, human primary T lymphocytes	Inhibit ERK1-2/NF-κB signaling pathway, reduce IL-6, IL-1β, TNF-α and other pro-inflammatory factors, enhance anti-inflammatory molecules	It can exert anti-inflammatory effect effectively *in vivo*and vitro	Raimondo et al. (2022)
Lotus	Induced cell inflammation model, cell migration model	Reduced the ability to induce inflammation by LPS and reduced the concentration of nitrite in the culture medium	It has the potential of wound healing and can promote cell migration *in vitro*	Lo et al. (2025)
Kiwifruit	Targeted delivery model	It has natural liver targeting, reduces drug leakage in the gastrointestinal tract, and enhances intestinal epithelial permeability	Realize the aggregation of liver-specific drugs and significantly inhibit the proliferation of abnormal cells	Fang et al. (2023)
Bitter melon	Drug resistance model	Significantly reduced the expression of NLRP3 inflammatory body	It provides a new strategy to overcome the antibacterial resistance in oral drug delivery system	Yang et al. (2021)
Grape	Drug delivery models	Highly loaded and delivered HSP70 to human cells	It shows great potential as a functional molecular delivery carrier	Kilasoniya et al. (2023)
Petasites japonicus	Dendritic cells (DCs), initial T cells	Promote DC maturation (upregulate CD80, CD86, MHC-I, MHC-II), enhance antigen presentation capacity, reduce antigen uptake; activate initial T cells to differentiate into Th1 and cytotoxic CD8^+^ T cells	As a new type of immune adjuvant, it has strong immune stimulating characteristics and is used in immunotherapy	Han et al. (2021)
Mulberry bark	Mouse colitis model	The AhR signaling pathway was activated by HSPA8	It has a protective effect on colitis and may be used to prevent and treat intestinal related inflammatory diseases	Sriwastva et al. (2022)
Ginseng	POF model	Rich in saponins and other antioxidant and anti-apoptotic compounds, which may inhibit oxidative stress by activating PI3K/AKT signaling pathway	Reduce granulosa cell apoptosis, enhance follicle survival, delay ovarian dysfunction	Chu et al. (2024), Kim et al. (2023b)
Ginger	Endometrial repair model	Upregulate VEGF and IGF-1 expression	Promote angiogenesis and endometrial repair, improve thin endometrium	Kumar et al. (2022), Zhu and He (2023)
Turmeric	High-fat diet mouse model	By promoting lipolysis, inhibiting fat production, inducing browning of adipocytes and triggering apoptosis in various ways after endocytosis	Anti-obesity effect, improve the probability of conception in PCOS patients	Wang et al. (2024b)
Grapefruit	Ovarian and endometrial inflammation model	Rich in flavonoids, regulate TNF-α, IL-6 and other pro-inflammatory factors expression; inhibit NF-κB inflammatory pathway	Improve ovarian microenvironment; reduce endometrial inflammatory response	Iriawati et al. (2024), Chen et al. (2023b), Wang et al. (2014)
Tea	Endometritis model	Contains natural anti-inflammatory components and inhibits NF-κB pathway	Effectively reduce endometrial inflammatory response	Iriawati et al. (2024), Chen et al. (2023b)
Pomegranate	Male infertility model (sperm)	Rich in polyphenolic compounds, effective in clearing ROS	Reduce sperm DNA breakage and improve sperm quality	Kim et al. (2023c), Nguyen et al. (2023)
Blueberry	Male infertility model (sperm)	Rich in polyphenolic compounds, effective in clearing ROS	Reduce sperm DNA breakage and improve sperm quality	Kim et al. (2023c), Nguyen et al. (2023)
Ginseng	Male infertility model (sperm)	Enhance mitochondrial function	Improve sperm vitality	Ni et al. (2022)
Ginseng	Early embryos	Simulate the microenvironment of the body and provide natural growth factors	Support early embryonic development	Shumakova and Kiselev (2014)
Mulberries, Grapefruit	Embryo culture process	Rich in antioxidants to reduce ROS accumulation	Improve the blastocyst formation rate	Garrett et al. (2024),Feng et al.; Savcı et al. (2021)
Cactus fruits	Male infertility model (sperm)	Contains plant sphingolipid components to stabilize sperm membrane structure	Reduce freezing recovery damage and protect sperm membrane integrity	Valentino et al. (2024)
Sesame	Male infertility model (sperm)	Regulate calcium ion channels and promote sperm activation	Improve fertilization rates	Zhao et al. (2025)
Summary of PDEVs versatility	Reproductive system, gut flora, immune system	1. Carrier Function: The natural lipid bilayer protects its contents, exhibiting excellent biocompatibility, low immunogenicity, and cross-species transferability. It can carry nucleic acid drugs (siRNA, mRNA) or small molecule drugs for targeted delivery. 2. Immune Regulation: Modulates macrophage polarization (e.g., promoting M2 phenotype), regulates T cell subpopulation ratios, and induces immune tolerance. 3. Microbiota Regulation: May influence the composition of reproductive tract microbiota, indirectly modulating local immunity and nutritional environment. 4. Induce apoptosis and promote endometrial decidualization 5. Hormone replacement therapy to replace or supplement traditional estrogen regimens	1. Enhances oral drug bioavailability, prevents gastrointestinal degradation, and facilitates intestinal epithelial barrier penetration. 2. Possesses potential significance in maintaining maternal-fetal immune balance and preventing embryonic rejection. 3. Regulates intestinal homeostasis, inflammatory responses, and obesity-related metabolic disorders with their complications, making it applicable for treating obesity-associated reproductive disorders such as PCOS. 4.Improves endometrial receptivity. 5.Reduce hormone dependence and reduce the risk of OHSS.	

## 5 Discussion

EVs, as a novel molecular messenger system in reproductive medicine, are undergoing a critical transition from basic exploration to clinical translation (Bai et al., 2024; Cong et al., 2022). Recent studies have revealed that these nanoscale carriers not only participate in the regulation of the embryo implantation microenvironment, follicle development synchronization, and sperm maturation through the delivery of active substances such as miRNAs, proteins, and lipids, but also play a pivotal role in molecular regulation mechanisms related to endometriosis, premature ovarian failure, and male infertility. Notably, liquid biopsy techniques based on EVs have shown potential to break through traditional invasive diagnostic methods (Kumar et al., 2024). In terms of technological innovation, the integration of microfluidic chips and artificial intelligence is reshaping the purification technology system for EVs (Guo et al., 2018; Nicoliche et al., 2020). These technological breakthroughs have laid the foundation for establishing standardized production systems, making targeted exosome therapy through engineering modifications possible (Tao et al., 2023; Marquez et al., 2024; Li N. et al., 2023). Simultaneously,The emergence of PDEVs represents a paradigm shift in reproductive medicine, marking the transition from traditional “animal-led” approaches to innovative “plant-enabled” strategies. This evolution addresses several critical limitations of conventional animal-derived extracellular vesicles (ADEVs), including ethical concerns, potential zoonotic risks, and batch-to-batch variability. PDEVs offer distinct advantages in terms of biocompatibility, stability, and the ability to deliver plant-specific bioactive compounds that are absent in animal systems (Song et al., 2025; Dutta et al., 2025).

As the field progresses, PDEVs may not only complement existing ART protocols but potentially enable entirely new therapeutic strategies for reproductive disorders. Their plant origin offers a sustainable, scalable, and potentially safer alternative to current animal-derived products, aligning with the growing demand for natural and ethical medical solutions (Chen et al., 2022; Yazdanpanah et al., 2025). The transition from “animal-led” to “plant-enabled” reproductive medicine may well represent the next frontier in fertility treatment innovation.
